# Supplementary material for: Bacterial and Fungal Diversity Inside the Medieval Building Constructed with Sandstone Plates and Lime Mortar as an Example of the Microbial Colonization of a Nutrient-Limited Extreme Environment (Wawel Royal Castle, Krakow, Poland)
Source: Microorganisms. 2019 Oct 3;7(10):416. doi: 10.3390/microorganisms7100416 (PMC6843168; doi:10.3390/microorganisms7100416)
Supplement: Supplementary file 1 [file microorganisms-07-00416-s001.zip › microorganisms-606030-supplementary-2.pdf]

Table S1. Diversity and eveness indices for microorganisms detected in different sampling periods and material types.

	Ancient walls -sand-like/dust samples				Indoor air sampled onto agar plates			
	bacteria		fungi		bacteria		fungi	
	shannon	pielou	shannon	pielou	shannon	pielou	shannon	pielou
April	7.381356	0.830986	4.166877	0.446459	4.556695	0.740956	3.789885	0.646961
June	7.556157	0.767107	4.925773	0.491478	4.200664	0.769432	4.319733	0.650184
August	7.715185	0.764028	4.683442	0.450154	4.755116	0.714173	4.027622	0.676434
October	7.558044	0.744560	5.09555	0.512880	4.296659	0.673981	2.775719	0.502523

Table S2. Number of microorganisms detected in sand-like/dust samples and air sampled onto agar plates.

	April	June	August	October
**Bacterial diversity of sand-like/dust samples from ancient walls**				
**At family level**	142	199	232	240
**At genus level**	201	337	399	421
**Fungal diversity of sand-like/dust samples from ancient walls**				
**At family level**	181	238	266	226
**At genus level**	297	470	548	439
**Bacterial diversity of air sampled onto agar plates**				
**At family level**	23	11	22	28
**At genus level**	33	17	27	41
**Fungal diversity of air sampled onto agar plates**				
**At family level**	26	46	22	21
**At genus level**	34	60	33	34
**Bacteria detected in both sand-like/dust and air samples**				
**At family level**	13	11	20	26
**At genus level**	10	16	19	28
**Fungi detected in both sand-like/dust and air samples**				
**At family level**	23	44	22	21
**At genus level**	30	54	32	24

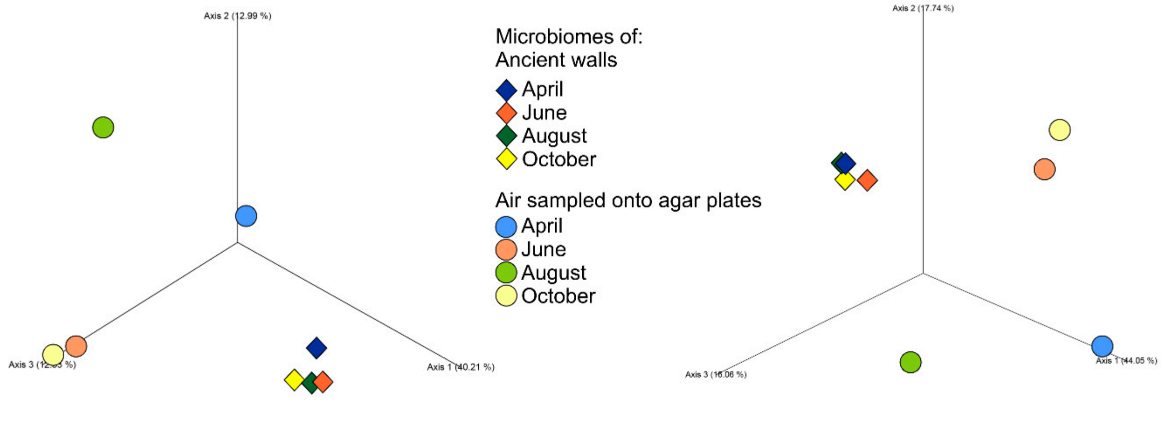

(a) (b)

Figure S1. Principal Coordinates Analysis (PCoA) of Bray–Curtis dissimilarity for: (**a**) bacteria and (**b**) fungi

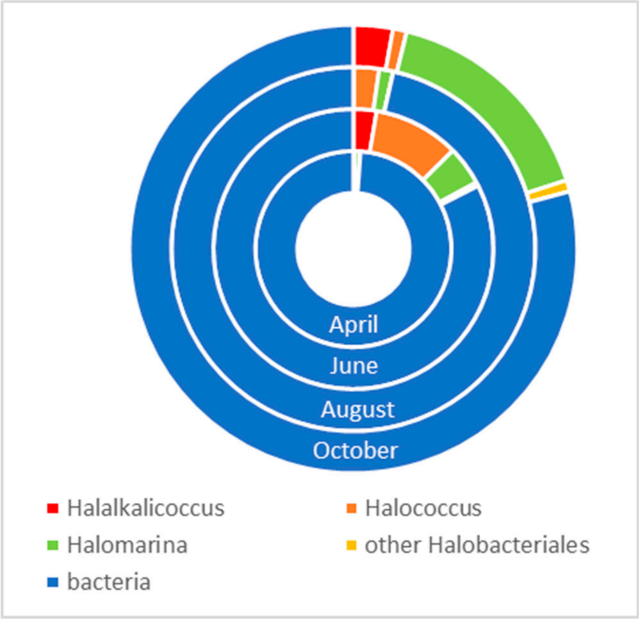

Figure S2. Microbial community structure for samples collected from ancient walls, including unfiltered archaeal sequences of amplicons of 16S rDNA.
