# Supplementary figures and images for: Bacterial and Fungal Diversity Inside the Medieval Building Constructed with Sandstone Plates and Lime Mortar as an Example of the Microbial Colonization of a Nutrient-Limited Extreme Environment (Wawel Royal Castle, Krakow, Poland)
Source: Microorganisms. 2019 Oct 3;7(10):416. doi: 10.3390/microorganisms7100416 (PMC6843168; doi:10.3390/microorganisms7100416)

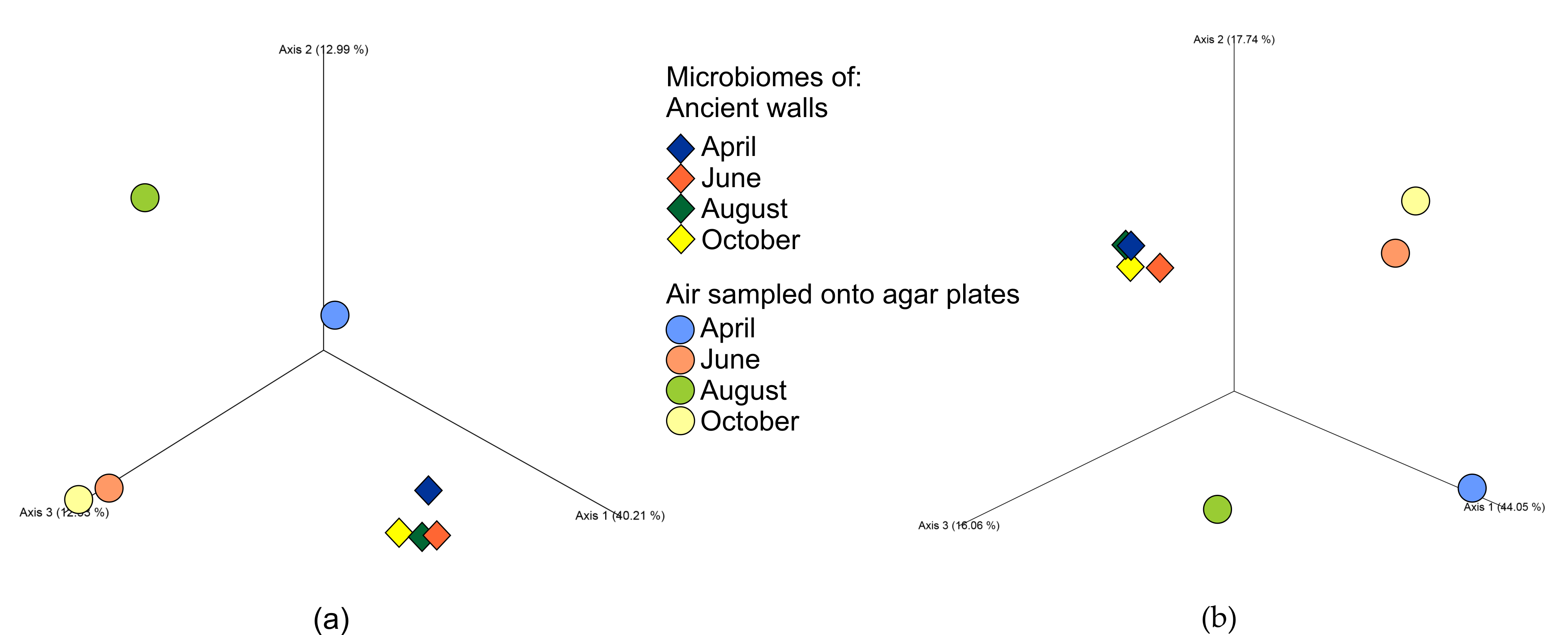

Supplement: Supplementary file 1 [file microorganisms-07-00416-s001.zip › Figure S1.tif]

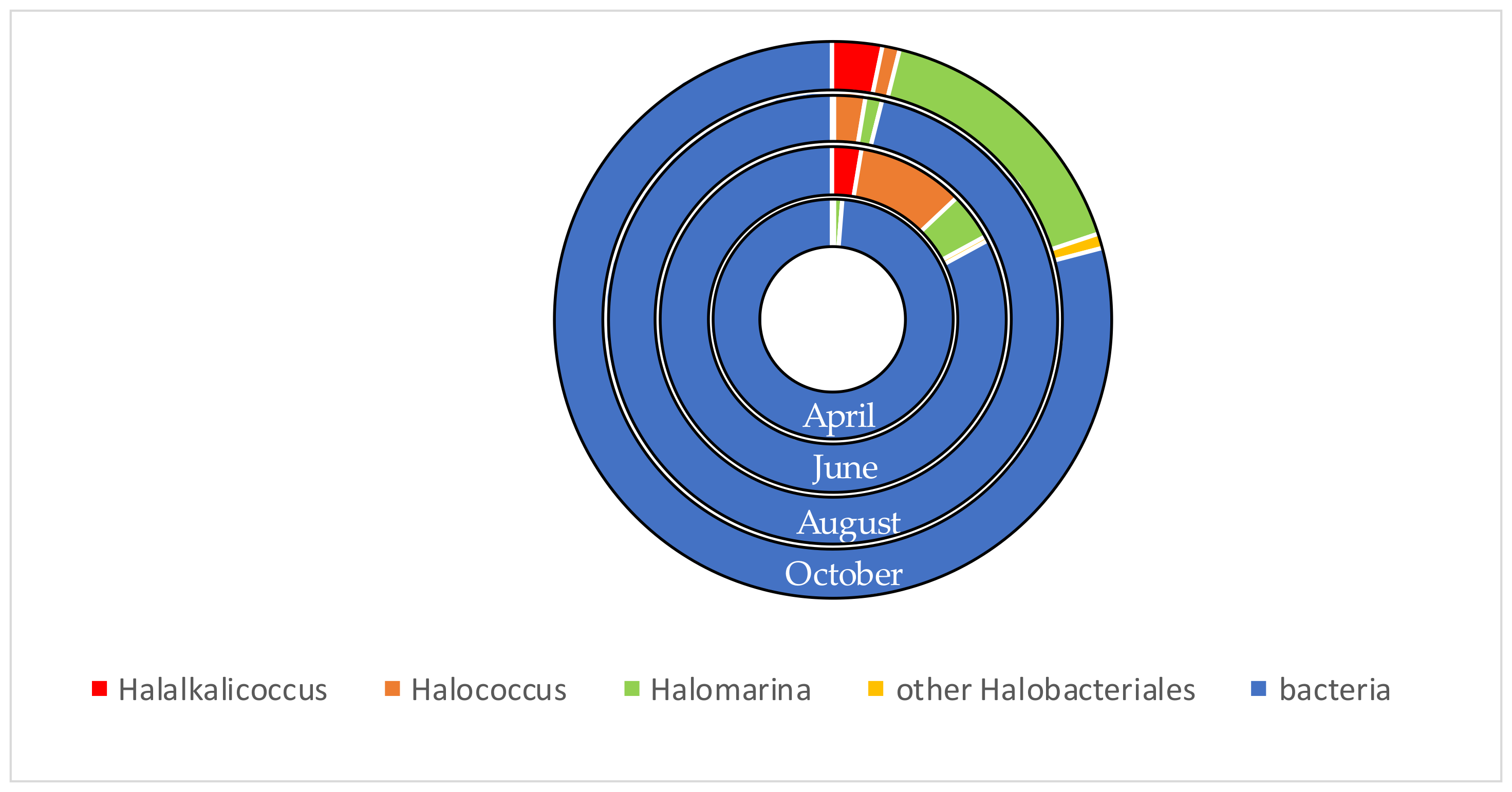

Supplement: Supplementary file 1 [file microorganisms-07-00416-s001.zip › Figure S2.tif]
